# Mental health outcomes following COVID-19 infection: impacts of post-COVID impairments and fatigue on depression, anxiety, and insomnia — a web survey in Sweden

**DOI:** 10.1186/s12888-022-04405-0

**Published:** 2022-11-29

**Authors:** Farzaneh Badinlou, Tobias Lundgren, Markus Jansson-Fröjmark

**Affiliations:** Department of Clinical Neuroscience, Centre for Psychiatry Research, Karolinska Institutet, and Stockholm Health Care Services, Region of Stockholm, Norra Stationsgatan 69, 113 64 Stockholm, Sweden

**Keywords:** COVID-19, Mental health, Depression, Anxiety, Insomnia, Post-COVID, Fatigue, Motivation

## Abstract

**Background:**

The negative impact of the COVID-19 pandemic on the mental health is now clearly established. However, information on the levels of mental ill health of people infected with COVID-19 and potential correlates of poor mental health is still limited. Therefore, the current study aimed to study indicative of potential mental health problems in individuals with a history of probable or confirmed SARS CoV-2 infection/infections and address the impacts of post-COVID impairments and fatigue following COVID-19 infection/infections on depression, anxiety, and insomnia.

**Methods:**

A web-survey including demographics, questions related to COVID-19 status and post-COVID impairments, and standardized measures of depression, anxiety, insomnia, and fatigue was completed by 507 individuals with a history of probable or confirmed SARS CoV-2 infection/infections.

**Results:**

We found significant rates of significant depression, anxiety, and insomnia in our sample, with more than 70% experiencing levels above the clinical cut offs for at least one psychological health problems. Higher levels of depression, anxiety, and insomnia were associated with the severity of COVID-19 infection in the acute phase, hospitalization because of COVID-19, and higher levels of post-COVID impairments and fatigue. Reduced motivation emerged as the strongest predictor for mental ill health.

**Conclusions:**

These findings highlight that individuals infected with COVID-19, especially those who still have experienced post-COVID impairments, are more likely to suffer from mental ill-health and may be more vulnerable for poor mental health outcomes. Therefore, more effective actions are needed to take in order to promote and protect mental health of individuals with a history of COVID-19 infection.

## Background

Since the beginning of the COVID-19 pandemic, the study of mental health has been the focus for many investigations around the world. Early studies focused mainly on the psychosocial response to the COVID-19 pandemic and impacts of pandemic-related strategies, such as quarantine, lockdown, closing schools/universities, movement and travel restrictions, on mental health outcomes in the general population [1–6]. Findings from studies in different countries showed that the prevalence of mental health problems increased significantly in the general population during the COVID-19 pandemic [7–11]. In Sweden, studies of mental health in the early phase of the COVID-19 pandemic showed elevated rates of depression, anxiety, and insomnia in the general population compared with before the pandemic, the same as in other counties [12, 13].

With increasing number of COVID-19 cases and hospitalization due to COVID-19 in the different countries, a possible bidirectional association between COVID-19 and mental health problems was attracting the attention of researchers [14–16]. On the one hand, pre‐existing mental health problems could increase the risk to get COVID infection, or make the outcomes of the infection worse [17–21]. On the other hand, a significant proportion of patients with and survivors of COVID-19 reported numbers of psychological issues, such as post-traumatic stress disorder (PTSD), depression, anxiety, insomnia, and obsessive–compulsive symptoms [22–26].

Follow-up studies on COVID-19 survivors after discharge from hospital or/and recover from COVID-19 reported an unexpected result called post COVID-19 condition [27–29]. This condition affects individuals infected with COVID-19 and is characterized by a wide range of persistent symptoms even after months of the onset of disease or hospitalization [28, 30–35]. The post COVID-19 condition has also been widely reported by individuals with the mild-to-moderate symptoms in the acute phase of SARS-CoV-2 disease, mostly recovered without requiring special treatment or/and hospitalization [36]. A recent meta-analysis, including data from fifty studies, shows that the global prevalence of post-COVID condition was 0.43 (95% confidence interval [CI], 0.39–0.46). In other words, nearly half of the people with a history of probable or confirmed SARS CoV-2 infection experienced mid- and long-term symptoms after recovery from COVID-19 [37]. Long‐term impacts of COVID‐19 infection can be multi-systemic problems and disabilities, and fatigue in general was the most commonly reported symptom in previous studies [37–42].

A collaborative cross-sectional study in Japan and Sweden examining associations between mental health and post-COVID conditions showed that post-COVID condition includes mental health issues, such as depression and anxiety, were significantly higher in groups with post-COVID conditions, compared to noninfected groups and infected group without post-COVID conditions. In addition, post-COVID conditions was demonstrated to be a risk for developing mental health illness [43]. Another study, conducted in Sweden reexamined the prevalence of depression, anxiety, and insomnia in the general population and the impact of COVID-19-related persistent symptoms on these psychiatric symptoms two years after the first appearance of COVID-19. Findings showed that the level of depression did not change significantly, the level of anxiety decreased, and the level of insomnia had increased compared with the early phase of the pandemic in Sweden. COVID-19-related persistent symptoms were found as an important vulnerability factor for mental ill-health [44]. However, research on the mental health of people infected with COVID-19 is still limited, and it is not clear how post-COVID impairments and fatigue, as the most common persistent symptom, might interrelate with mental health outcomes. The first purpose of the current study was to determine indicative of potential mental health problems in individuals with a history of probable or confirmed SARS CoV-2 infection/infections. To further enhance the knowledge, the second aim of the study was to address the impacts of post-COVID impairments and fatigue on mental health outcomes following COVID-19 infection/infections.

## Methods

### Participants and setting of the study

A total 507 people was included in the current study. Inclusion criteria were: (*i*) infected with COVID-19; (*ii*) age (≥ 18 years); (*iii*) being a resident of Sweden; (iv) ability to understand Swedish and use the internet in order to complete web-survey. The following exclusion criteria was used; (*i*) age under 18 years old, (*ii*) did not complete scales, or (*iii*) not infected with COVID-19. They were therefore unable to complete COVID-19-related and post-COVID questions. We used convenience sampling to recruit participants in the current study. In that, an announcement including information about the study and a link to a web-survey was posted in COVID-19-related Facebook groups and sites, and the Karolinska institute homepages. In order to recruit potential participants, the study information was administered at four primary care centers treating post-COVID patients in Stockholm, Sweden.. The participants’ age ranged between 19 and 81 years old (M = 47.72, SD = 10.62). Demographic information about the participants is presented in Table 1.

**Table 1:** Sample characteristics (*N* = 507)

	n	%
Demographic variabels		
Gender		
Female	446	88
Male	61	12
Education		
Pre-secondary	14	2.8
Secondary	140	27.6
University/Post graduate	353	69.6
Marital status		
Single	106	20.9
Married	213	42
In a relationship	144	28.4
Divorced/separated/widowed	44	8.7
Work status		
Working full time/part time	335	66.1
Unemployed/unpaid work	23	4.5
Retired	24	4.7
Parental leave	5	1
Sick leave	107	21.1
Student	13	2.6
Economic status		
Below average	84	16.6
Average	247	48.7
Above average	176	34.7

Table 2 presents the descriptive statistics of COVID-19-related variables. The majority of the participants reported they had been infected with COVID-19 for the first time in the first and second wave of COVID-19 in Sweden, spring and autumn 2020 [45].

**Table 2:** Descriptive statistics of COVID-19-related variables

	n	%
Infected with COVID-19		
Yes, one time	316	62.3
Yes, two times	105	20.7
Yes, more than two times	12	2.4
Yes, unconfirmed	74	14.6
Infected with COVID-19 for the first time		
First wave of COVID-19 in Sweden, spring 2020	160	38.4
Second wave of COVID-19 in Sweden, autumn 2020	88	21.1
During the year 2021	95	22.8
During the year 2022	74	17.7
Hospitalization for COVID-19		
Yes	52	12.5
No	363	87.5
Being a high-risk group for COVID-19		
Yes	98	19.6
No	403	80.4
Vaccinated against COVID-19		
Yes, one dose	21	4.1
Yes, two doses	157	31
Yes, three doses	243	47.9
No	86	17

### Measures

#### Demographic information

Demographic variables were age, gender, education, marital status, work status, and economic status.

#### COVID-19-related variables

COVID-19-related variables included infected with COVID-19, hospitalization due to COVID-19, been high-risk group for COVID-19, vaccinated against COVID-19, and COVID-19 severity in the acute phase. Infected with COVID-19 was measured by a single item in which respondents stated on a 4-point scale if they have/had a confirmed COVID-19 infection supported by positive tests for COVID-19 virus (PCR) and/or positive rapid antigen test on a 4-point scale (yes, I have had it one time, yes, I have had it two times, yes, I have had it more than two times, yes, I believe I've had it, but have not had it confirmed). Respondent also were asked to state on a binary scale (yes, no) if they have/had been hospitalized because of COVID-19, high-risk group for COVID-19 (including high blood pressure, angina, stroke, heart disease, diabetes, cancer, smoking, respiratory diseases, and impaired immune system), and vaccinated against COVID-19. COVID-19 severity in the acute phase was measured with 15 items, including fever, fatigue, cough, loss of smell/taste, difficulty breathing/shortness of breath, headache/migraine, aches or pain in body, diarrhea, rash on skin, runny or blocked nose, nausea/vomiting, arrhythmia/palpitations*,* sore throat, cognitive difficulties such as memory and attention difficulties, and mental health issues such as sleep problems, depression and anxiety [46, 47]. Here participants rated symptoms that they had at the beginning of the infection or infections and those the following 4 weeks on a 4-point scale (0 = no, 1 = mild, 2 = moderate, 3 = severe) considering acute COVID-19 usually can last up to 4 weeks from the onset of symptoms [48]. The respondents’ answers to the 15 symptoms of COVID-19 items were summed up to calculate a COVID-19 severity in the acute phase (range 0—45, α = 0.77).

#### Post-COVID impairments

The post-COVID impairments in the current study were assessed with 54 items used in our previous study [38]. The items were based on previous questionnaires developed to examine long-term impacts of COVID-infection such as the Functional Compass COVID-19 questionnaire and the Long COVID Symptom and Impact Tools [36, 49] and comprehensive literature review of the long-term effects of COVID-19 [32, 33, 48, 50, 51]. Participants rated their impairments due to COVID-19 infection/infections on a 4-point scale (0 = no, 1 = mild, 2 = moderate, 3 = severe). Items were categorized into four sub-categories according to the International Classification of Functioning, Disability and Health [52] as impairments in mental functions including impaired orientation, brain fatigue, lack of appetite, sleep problems, concentration difficulties, attention difficulties, memory problems, impaired organization and planning, impaired mental functions of language, depression, anxiety, stress, obsessions, and compulsions; impairments in sensory functions and pain including poor quality of vision, dry/red/itchy eyes, ringing in ears or tinnitus, dizziness, disturbed balance, loss of taste, loss of smell, feeling of numbness/tingling, generalized pain, pain in head, chest pain, pain in stomach or abdomen, joint pain, and pain in multiple body parts; impairments body system functions including voice problems, impaired heart functions, respiratory distress, cough, tiredness or lack of energy (physical), shortness of breath, sore throat/difficult to swallow, vomiting, impaired nutrient uptake, diarrhea, weight change, nausea, fever/feeling of fever, chills/the feeling of freezing, impaired sexual desire and functions, impaired mobility of movement, decreased muscle power, movement problems, skin changes, rash, and hair loss; and impairments in activities and participation including difficulty taking care of yourself, impaired control of other diseases and drugs, keep special diet, and difficulties in doing housework, impaired work ability/study ability, and difficulty being to leisure activities. The respondents’ answers to each sub-category of post-COVID impairments were summed up and divided the number of items in order to obtain mean for each sub-category as a common way to analysis scores from observational measures in social sciences [53].

#### Fatigue

The Multidimensional Fatigue Inventory (MFI) is self-report instrument to measure fatigue. The MFI is a 20-item scale and consists of five subscales: general fatigue (GF), physical fatigue (PF), reduced motivation (RM), reduced activity (RA), and mental fatigue (MF). Each scale contains four items. The items are rated on 5-point scale, from 1 (Yes, that is true) to 5 (No, that is not true), scores range from 0 to 20 [54]. This test yields a total score (the sum of all items) and five scale scores calculated as the sum of the items within each subscale. Higher scores indicate higher fatigue levels. The subscale score > 12 was considered clinically significant fatigue [55], and the total score > 60 was considered clinically significant multidimensional fatigue [56]. In this study, we used the Swedish version. Psychometric studies show that the Swedish version of the instrument is reliable and valid [57, 58].

#### Mental health variables

Mental health variables were considered as outcome variables in the current study and consisted of depression, anxiety, and insomnia. Depression was measured with the Patient Health Questionnaire-9 (PHQ-9). The PHQ-9 consists of nine items answered on a four-point scale (0–3), with the total score ranging from 0 to 27 [59, 60]. Anxiety was assessed with the General Anxiety Disorder-7 (GAD-7). The GAD-7 includes seven items answered on a four-point scale (0–3), scores range from 0 to 21 [61–64]. Insomnia was measured with the Insomnia Severity Index-7 (ISI-7). The ISI-7 consists of seven items to assess the nature, severity, and impact of insomnia answered on a five-point scale (0–4), the total score ranges from 0 to 28 [65, 66]. We used the recommended cutoff scores of ≥ 10 for detecting the clinically significant depression, anxiety, and insomnia in the current study [59, 64, 66].

### Procedure

The study was approved by the Swedish national ethical board (dnr 2021–06,617-01) and informed consent was obtained from all participants. Participants answered the survey in the online platform, Research Electronic Data Capture (REDCap), hosted locally at Karolinska institute [67, 68]. Data were collected between the 23^th^ of February and the 5^th^ of April 2022.

### Statistical analysis

The data were analyzed using IBM SPSS (IBM, Version 26) [69] and checked for normality, missing values, outliers, or any errors in data. The mean, standard deviation, and numbers or percentage were calculated for each continuous and categorical variable. For purposes of analysis, categorical variables were dichotomized; education (dichotomous: high education, low education), marital status (dichotomous: in relationship, not relationship), work status (dichotomous: not working, working), economic status (dichotomous: above average, average and below average), and infected with COVID-19 (dichotomous: confirmed COVID-19 infection, not confirmed COVID-19 infection). Zero-order correlations (Pearson's *r*) were calculated to examine the relationship among all measures and to identify primary predictors of the model. To address the second aim, hierarchical linear regression models were applied to explore the contribution of potential predictors on mental health outcomes. Three separate analyses were employed to estimate the predictors’ association with depression, anxiety, and insomnia as dependent variables. For all three regression models, the significant variables were considered as potential predictors. Due to performing multiple tests on a single dataset, alpha-level was adjusted to *p* ≤ *0.01* in the current study.

## Results

### Descriptive statistics for mental health, fatigue, and post-COVID impairments variables

Table 3 presents descriptive statistics for the measures of depression, anxiety, and insomnia. In that, 55%, 20.5%, and 60.9% of the participants reached the cutoff for clinical depression, anxiety, and insomnia, respectively. The majority of the participants reached cutoff for at least one psychological health problems (74.7%). Intercorrelations between these measures were *r* = 0.71 for depression and anxiety scores, *r* = 0.58 for depression and insomnia scores, and *r* = 0.58 for anxiety and insomnia scores.

**Table 3:** Descriptive statistics for the PHQ-9, GAD-7, and ISI-7

Outcome	α	Mean (SD)	Range	n (%)
Depression (PHQ-9)	0.80	10.78 (5.7)		
Minimal			0–4	66 (14.2)
Mild			5–9	143 (30.8)
Moderate			10–14	141 (30.4)
Moderately severe			15–19	76 (16.4)
Severe			20–27	38 (8.2)
Anxiety (GAD-7)	0.84	6.12 (4.89)		
None			0–4	211 (44.6)
Mild			5–9	165 (34.9)
Moderate			10–17	75 (15.9)
Severe			15–21	22 (4.7)
Insomnia (ISI)	0.88	11.87 (6.82)		
None			0–7	141 (29.9)
Sub-threshold			8–14	161 (34.2)
Moderate			15–21	126 (26.8)
Severe			22–28	43 (9.1)

α = Cronbach's alpha

Table 4 includes summary results from the fatigue scores and prevalence rates per domain. Of all participants, 94.7% (*n* = 481) reached cutoff for severe fatigue on at least one fatigue dimension, based on cutoff scores > 12. For more details, 89.9% (*n* = 456), 85.6% (*n* = 434), 84.8% (*n* = 430), 51.6% (*n* = 255), and 73.4% (*n* = 372) met criteria for likely clinically significant general fatigue, physical fatigue, reduced activity, reduced motivation, and mental fatigue, respectively. Of all participants, 39.3% (*n* = 194) reached the cutoff for clinically significant multidimensional fatigue. As seen in Table 4, these measures are intercorrelated (see Table 4).

**Table 4:** Descriptive statistics and correlations for the subscales of the MFI-20

	α	Mean	SD	1	2	3	4	5
1.General fatigue	0.77	17.2	3.43	-	0.76*	0.70*	0.41*	0.49*
2.Physical fatigue	0.78	16.65	3.59		-	0.75*	0.40*	0.48*
3.Reduced activity	0.75	16.05	3.67			-	0.46*	0.60*
4.Reduced motivation	0.81	12.51	4.17				-	0.50*
5.Mental fatigue	0.77	14.44	3.91					-

α = Cronbach's alpha

^*^ *p* < 0.01

Those surveyed also provided data on post-COVID impairments, each on a scale from 0 to 3, higher scores indicating higher levels of impairments. Table 5 includes summary results from the post-COVID impairments. The highest level of impairment was impairments in activities and participation, followed by impairments in mental functions. As seen in Table 5, these variables are intercorrelated (see Table 5).

**Table 5:** Descriptive statistics and correlations for post-COVID impairment variables

	α	Mean	SD	1	2	3	4
1. Impairments in mental functions	0.90	1.31	0.67	-	0.69*	0.68*	0.66*
2. Impairments in sensory functions and pain	0.88	1.11	0.66		-	0.81*	0.62*
3. Impairments in body system functions	0.90	1.03	0.61			-	0.72*
4. Impairments in activities and participation	0.84	1.31	0.81				-

α = Scale Internal Consistency

^*^ *p* < 0.01

### Correlational analyses

Table 6 presents correlations between the outcome variables with participant background, COVID-19-related factors, post-COVID impairments, and fatigue dimensions. Notably, the background variables failed to significantly correlate with the mental health outcomes. More serious condition in the acute phase of COVID-19 infection was significantly related with all three outcome variables. Hospitalization because of COVID-19 achieved significant but small correlation with insomnia. Post-COVID impairments and fatigue variables significantly positively correlated with depression, anxiety, and insomnia.

**Table 6:** Correlations between potential predictors and depression, anxiety, and insomnia

Potential Predictors	Depression (PHQ-9)	Anxiety (GAD-7)	Insomnia (ISI-7)
Background variables			
Age	-0.06	-0.145	0.104
Gender	0.011	0.031	0.026
Education	-0.095	-0.041	-0.111
Marital status	-0.056	0.021	-0.071
Work status	0.143	0.101	0.065
Economic status	0.077	0.031	0.001
COVID-19-related variables			
Infected with COVID-19 (Confirmed)	0.030	0.141	0.101
Severity of COVID-19 infection	0.343*	0.227*	0.288*
Been high-risk group	-0.084	-0.061	-0.110
Hospitalization for COVID-19	-0.101	-0.036	-0.166*
Vaccinated against COVID-19	-0.035	-0.064	-0.042
Post-COVID impairments			
Impairments in mental functions ^a^	0.547*	0.361*	0.342*
Impairments in sensory functions and pain	0.392*	0.417*	0.473*
Impairments in body system functions	0.417*	0.218*	0.337*
Impairments in activities and participation	0.473*	0.257*	0.307*
Fatigue variables			
General fatigue	0.510 *	0.262*	0.365*
Physical fatigue	0.461*	0.204*	0.286*
Reduced activity	0.532*	0.265*	0.297*
Reduced motivation	0.568*	0.402*	0.329*
Mental fatigue	0.514*	0.340*	0.232*

^*^ *p* < 0.01

a Adjusted to exclude depression, anxiety, stress, or sleep problems

### Regression analyses

Table 7 presents the final results of the hierarchical regression models for depression, anxiety, and insomnia. In a three-block model, COVID-19-related factors including severity of COVID-19 infection in the acute phase and hospitalization for COVID-19 were entered into the first block, followed by post-COVID impairments including impairments in mental functions, sensory functions and pain, body system functions, and activities and participation, entered in block 2, and fatigue factors including general fatigue, physical fatigue, reduced activity, reduced motivation, and mental fatigue, entered in block 3. All three blocks reached statistical significance for each of the outcome variables. For depression, fatigue accounted for the largest part of the variance, with $\Delta$ R^2^ accounting for 23% of the variance. For anxiety, both fatigue factors and post-COVID impairments accounted similar variance in the equations, with $\Delta$ R^2^ accounting for 12% of the variance. For insomnia, COVID-19-related factors accounted for the largest part of the variance. Interestingly, out of all variables, reduced motivation came out as the most important predictor for all three mental health outcomes.

**Table 7:** Results from hierarchical multiple regression analyses of depression, anxiety, and insomnia

	**Depression**		**Anxiety**		**Insomnia**	
**Block 1**	**ΔR2 = 0.144, F = 16.08, *p* < 0.001**		**ΔR2 = 0.071, F = 7.46, = 0.001**		**ΔR2 = 0.130, F = 14.59, *p* < 0.001**	
	β	p	β	p	β	p
Severity of COVID-19 infection	0.144	0.035	0.125	0.134	0.174	0.038
Hospitalization for COVID-19	0.033	0.528	0.049	0.440	0.082	0.199
**Block 2**	**ΔR2 = 0.165, F = 11.14, *p* < 0.001**		**ΔR2 = 0.119, F = 7.003, *p* < 0.001**		**ΔR2 = 0.062, F = 3.67,** ***p* = 0.007**	
Impairments in mental functions ^a^	0.243	0.014	0.291	0.017	0.037	0.764
Impairments in sensory functions and pain	0.082	0.393	0.212	0.074	0.018	0.880
Impairments in body system functions	0.050	0.624	0.033	0.794	0.093	0.467
Impairments in activities and participation	0.054	0.519	0.142	0.165	0.128	0.219
**Block 3**	**ΔR2 = 0.230, F = 18.11, *p* < 0,001**		**ΔR2 = 0.119, F = 6.36, *p* < 0.001**		**ΔR2 = 0.091, F = 4.69, *p* < 0.001**	
General fatigue	0.099	0.157	0.019	0.810	0.188	0.024
Physical fatigue	0.127	0.091	0.046	0.612	0.019	0.837
Reduced activity	0.016	0.843	0.115	0.247	0.177	0.077
Reduced motivation	0.448	< 0.001	0.395	< 0.001	0.286	< 0.001
Mental fatigue	0.010	0.897	0.019	0.841	0.036	0.713

a Adjusted to exclude depression, anxiety, stress, or sleep problems

## Discussion

The first aim of the current study was to examine levels of mental health problems following COVID-19 infection/infections. The second aim was to study associations between COVID-19-related factors, post-COVID impairments as well as fatigue and mental health problems in individuals with a history of probable or confirmed SARS CoV-2 infection/infections. Results showed significant rates of significant depression (55%), anxiety (20.5%), and insomnia (60.9%) in our sample. The correlational analyses show that severity of COVID-19 infection in the acute phase and hospitalization due to COVID-19 were significantly correlated with mental health outcomes. All post-COVID impairments and fatigue dimensions were significantly associated with depression, anxiety, and insomnia. In multivariate models, generally, severity of COVID-19 infection in the acute phase explained a portion of the variance in depression and insomnia and impairments in mental functions explained variance in depression and anxiety. However, reduced motivation emerged as the most consistent predictor of all three mental health outcomes.

In our study, rates of mental health problems were clearly higher than previous studies using the same measures and cut-off levels, noticeably in depression and insomnia. There are a number of likely explanations. The first possible explanation for these findings lies in our sample and inclusion criteria. In the previous studies, the participants were recruited from social media and age ≥ 18 was a main inclusion criterion [12, 43, 44], whereas we used convenience sampling targeting individuals with a history of probable or confirmed COVID-19 infection. As a result, in studies by Matsumoto et al. [43] and Brocki et al. [44], 13% and 51.2% of the participants reported that they have been infected with the COVID-19, respectively, whereas in our study, 85% of the participants reported confirmed COVID-19 infection/infections supported by positive tests for COVID-19 virus (PCR) and/or positive rapid antigen test, including one time, 61.9%, two times, 20.7%, and more than two times, 2.4%. The second possible explanation of these results is that diagnosis of COVID-19 is likely to be associated with increased rates of mental health problems and subsequent psychiatric diagnoses. In that, psychiatric illness and mental health problems such as depression, anxiety, insomnia, post-traumatic stress disorder, obsessive–compulsive symptoms, and substance use disorders are highly prevalent in patients diagnosed with COVID-19 [16, 21–26, 70, 71]. The third possible explanation is that a major proportion of participants in the current study had experienced the post-COVID impairments, such as impairments in mental function, sensory functions and pain, and body system functions. These findings are in line with a previous study that showed that incidence of clinically significant mental health problems was higher in COVID-19-infected participants, and it was more prominent in those with post-COVID conditions [43]. Furthermore, there are a number of previous studies indicating co-occurrence between pain, physical health problems and mental health illness [72–74]. The final possible explanation is the timing of data collection. Data for the current study was collected after the peak of the Omicron variant in Sweden, January 2022, which increased COVID-19 cases dramatically and tightened the restrictions. As a result, the rate of reinfection increased. In our sample, 19% (*n* = 99) of the participants reported that they had been infected for a second time and even a third time in January/February 2022. Considering possible explanations, we can safely say that individuals infected with COVID-19, especially those experiencing post-COVID impairments, are more likely to suffer from mental ill-health and may be more vulnerable for poor mental health outcomes.

Looking at potential predictors, prior severity of COVID-19 infection, post-COVID impairments and fatigue dimensions were associated with poor mental health outcomes and hospitalization because of COVID-19 was positively correlated with insomnia (see Table 6). These results are in line with previous studies showing high levels of mental health problems months after COVID-19 recovery, and a more serious COVID-19 infection in acute phase might be a risk factor for developing mental health illness [75, 76]. In more detail, individuals infected with COVID-19 showed the higher levels of mental health symptoms in the form of depression, generalized anxiety, and post-traumatic stress [43]. In more recent studies, post-COVID condition is clearly linked to mental ill-health [43, 76, 77].

Multivariate analyses were used to estimate the unique variance accounted for in depression, anxiety, and insomnia scores. The block of COVID-19-related variables explained unique variance in depression and insomnia and severity of COVID-19 infection appeared to contribute unique variance in depression and insomnia. The block of post-COVID impairments explained unique variance in depression and anxiety. The results are consistent with previous study reporting higher levels of mental health problems in individuals with post-COVID conditions compared with individuals who had developed COVID-19 without post-COVID conditions [43]. Looking at the importance of the separate variables within the set, impairments in mental functions appeared to contribute unique variance in depression and anxiety even we adjusted this variable to exclude depression, anxiety, stress, or sleep problems. It seems that impairments in mental functions such as impaired orientation, brain fatigue, concentration and attention difficulties, memory problem, and impaired organization and planning might be a risk factor for poor mental health outcomes. The block of fatigue variables, including general fatigue, physical fatigue, reduced activity reduced motivation, and mental fatigue, contributed unique variance in all three mental health outcomes. We further found that reduced motivation was interestingly the strongest predictors of risk for poor outcomes in depression, anxiety and insomnia. In other words, lower levels of motivation were associated with an increased risk of depression, anxiety, and insomnia symptoms. In the current study, motivation was assessed through four questions related to motivation dimension of the MFI-20 including “I feel like doing all sorts of nice things”, “I dread having to do things”, “I have a lot of plans”, and “I don’t feel like doing anything”. One possible explanation relies in that constancy in the face of difficulties as occurred during the COVID-19 pandemic is an important factor for lower wellbeing [78]. Life has been changed during the pandemic and a wide range of difficulties has been constantly experienced by many people such as financial difficulty, loneliness, fear of illness with COVID-19, loss of work, difficulties acquiring medication, difficulties accessing food, threats to personal safety, and difficulties in social functioning [9, 79, 80]. Therefore, reduced motivation could be a result of mental health issues or/and the pandemic period. Another possible explanation is that to stay motivated seems to play a key role for mental health outcomes in individuals infected with COVID-19. It has been nearly two years and a half since the COVID-19 pandemic started. People are tired to cope with pandemic and its consequences and demotivated to struggle with their post-COVID condition and improve their well-being beside the negative impacts on their life.

## Conclusion

In conclusion, the current study outlines that individuals with a history of probable or confirmed SARS CoV-2 infection/infections are more likely to suffer from mental health problems. Therefore, policies are needed in order to promote and protect mental health in the individuals infected with COVID-19 especially those who still have experienced post-COVID impairments.

### Implication

The identified factors that contribute to the level of mental health problems can be used for screening purposes in order to provide interventions for individuals infected with COVID-19 especially among those who still have experienced post-COVID condition. For example, the finding that reduced motivation was the strongest predictor of poor mental health suggests that staying motivated seems to be a core component in individuals infected with COVID-19 in order to manage their mental health. In the health care systems, the interventions targeting this group could use techniques like motivational interviewing in order to encourage patients to seek help, provide the initial motivation for treatment and increase likelihood of behavior changes [81–84].

### Limitations and future directions

There are several important limitations in the current study. First, using the cross-sectional design in this study did not enable us to interpret the findings as implying causality. Longitudinal studies are needed to identify how mental health outcomes change over time and the causal factors of changes. Second, we conducted this study on a convenience sample, which limits the generalizability of findings. Further studies are warranted to examine the mental health using more representative samples. Third, we gathered self-report data in this study. Self-report biases and poor recall should therefore be considered when interpreting the findings. Fourth, lack of baseline data related to mental health before infection/ infections did not enable us to examine confidently the impacts of post-COVID impairments and fatigue on mental health outcome. Fifth, lack of a control group of non-COVID affected subjects did not enable us to assess the weight of COVID infection versus psychosocial factors of the pandemic on mental health outcomes. Further studies are warranted to study mental ill health by comparing COVID affected and non-COVID affected subjects. Finally**,** the findings that reduced motivation and impaired mental functions were the strong predictor of poor mental health outcomes in the current study raise the chicken-and-the-egg dilemma. Further studies are vital to address the bidirectional associations between motivation as well as cognitive functions and mental health outcomes in individuals infected with COVID-19 especially those with post-COVID conditions.

## Data Availability

The datasets used and/or analyzed during the current study are available from the corresponding author on reasonable request.
